# African warthog buffy coat cells retain susceptibility to African swine fever virus following whole blood transportation and storage

**DOI:** 10.1186/s12985-025-03038-5

**Published:** 2026-01-09

**Authors:** Jessica Mason, Alyssa Deters, Erika Krueger, Lindsay Gabbert, Michael Puckette

**Affiliations:** 1 SAIC, Plum Island Animal Disease Center, P.O. Box 848, Greenport, 11944 NY USA; 2 Oak Ridge Institute for Science and Education, National Bio- and Agro- Defense Facility, 1980 Denison Ave, Manhattan, 66506 KS USA; 3 U.S. Department of Homeland Security, Science and Technology Directorate, Plum Island Animal Disease Center, P.O. Box 848, Greenport, 11944 NY USA; 4 U.S. Department of Agriculture, National Bio- and Agro-Defense Facility, 1980 Denison Ave, Manhattan, 66506 KS USA

**Keywords:** Buffy coat, African swine fever virus, Warthog, Wildlife, Hemadsorption, Swine, ASF, Blood

## Abstract

The spread of African Swine Fever (ASF) across much of the world is a profound challenge to both domestic and wild Suidae populations. While vaccine and countermeasure development for domestic swine are ongoing, the understanding of ASF within wildlife is limited. This gap in knowledge is largely due to the requirements of working with African Swine Fever Virus (ASFV) in high containment laboratories, the complexity of utilizing wildlife in these settings, and researcher access to non-native species. Researching the role of ASFV within wildlife primary cells, outside a vivarium, is currently limited by the need to utilize either terminal processing procedures, such as harvesting alveolar macrophages or bone marrow, or processes requiring large volumes of blood, such as peripheral blood mononuclear cell harvests. The limited availability and access restrictions of susceptible wildlife makes obtaining these samples difficult, especially when working with endangered wildlife species. Harvesting buffy coat fractions is independent of terminal methodologies and the need for large volumes of blood. A methodology to support ASFV replication has recently been demonstrated utilizing domestic swine blood derived buffy coat cells. Through establishing collaborations with zoological partners, we apply this methodology to small volumes of blood from an African Warthog, a known ASF susceptible species. These results demonstrate the value of such partnerships and how they may be utilized in the future to evaluate ASFV in wildlife derived cells in vitro.

**Supplementary Information:**

The online version contains supplementary material available at 10.1186/s12985-025-03038-5.

## Introduction

African Swine Fever Virus (ASFV), the causative agent of African Swine Fever (ASF), was first described in Kenya [1] and is traditionally endemic across eastern and southern Africa. Outbreaks of ASF outside of Africa have largely resulted from the transportation of infected swine or swine products including the most recent outbreak originating in 2007 in the Republic of Georgia [2] with subsequent spread into Europe, Asia, and the Caribbean [3–6].

While current ASFV research has largely focused on domestic swine, multiple wild Suidae species are susceptible to ASF [7–9] including non-threatened wildlife such as the African Warthogs (*Phacochoerus africanus*) and Red River Hogs (*Potamochoerus porcus*) [8, 9]; as well as populations of endangered wild Suidae such as the Visayan Warty Pig (*Sus cebifrons*) [8, 10]. Introduction of ASF into populations of endangered wild Suidae would have a devastating impact on species survivability and genetic diversity.

In the United States, an ASF outbreak may trigger the need to vaccinate small populations of these species to preserve captive breeding programs, similar to what was done with the California Condor (*Gymnogyps californianus*) in response to Highly Pathogenic Avian Influenza [11]. Decisions about vaccinating captive wildlife would depend on scientific data for that species. Usage of wildlife, such as African Warthogs, for in vivo studies carries additional difficulty and risk, relative to that of domestic swine. The requirement of a high-containment laboratory and use of a controlled pathogen, such as ASFV, amplify these complications. Maximizing experimental data that can be obtained in vitro is critical, as is utilizing data sets generated from non-terminal and low-risk sampling to preserve captive wildlife populations.

The lack of immortalized cell lines for many wildlife species and reliance of primary cells for culturing ASFV make understating the virus in wildlife difficult. Cultivation of ASFV remains largely dependent upon primary cells such as peripheral blood macrophages (PBMC) derived from blood [12–14] or pulmonary alveolar macrophages harvested from lungs [15, 16]. Porcine primary cells, particularly PBMCs, have subsequently been utilized to make attenuated viruses as vaccine candidates through the targeted deletion of viral genes [17–20]. Recently, results were published utilizing buffy coat cells obtained from small volumes of domestic swine blood as an alternative to the classical PBMC harvesting methodology [21]. Small volumes of blood are ideal when working with captive wildlife, as opportunities to obtain samples may be both limited and opportunistic in nature. Since African Warthogs serve as a virus reservoir [22] they represented an ideal species to evaluate the viability of this process for wildlife.

Through a partnership with zoological parks, whole blood collected in EDTA tubes from a captive Southern African Warthog (*Phacochoerus africanus sundevallii*) was obtained and resultant buffy coat cells were subsequently infected with ASFV. Warthog buffy coat (WBC) cells sustained replication of multiple ASFV isolates including an attenuated vaccine candidate. Successful buffy coat extraction and ASFV infection demonstrates the viability of whole blood transport for ASFV research in a high containment setting and provides a template for future evaluations of ASFV within wildlife samples.

## Methods

### Production of L929 conditioned media for use in M∅ base media

To provide secreted Granulocyte-macrophage colony-stimulating factor (GM-CSF) for differentiation of monocytes, NCTC clone 929 (L929) cell line (ATCC, CCL-1) was cultured in growth media (RPMI-1640 with HEPES/L-glutamine, 10% gamma-irradiated fetal bovine serum (FBS), and 1% antibiotic/antimycotic) until fully confluent. Following full confluence, supernatant was harvested and filtered through at 0.22 μm filter in duplicate. Filtered supernatant was aliquoted and stored at −20 °C until use.

### Production of ASFV georgia’07 spleen homogenate

Spleen homogenate stocks were generated from the spleen of an ASFV Georgia ’07 infected domestic swine. Spleen was weighed in a sterile dish after removing fatty and connective tissues, then transferred to a sterile frozen mortar and pestle set to be macerated. Macerated material was suspended 1 g/5 mL storage media (RPMI w 5% gamma-irradiated FBS, 2% antibiotic/antimycotic, 0.2% Gentamycin) and transferred into 50 mL conical tubes (Falcon^®^) for two freeze/thaw cycles at −70 °C. Supernatants were clarified by centrifugation at 205 x g for 20 min at 8 °C. Clarified supernatant was transferred into 50mL bottles for Sorvall™ Rotor SS-34 centrifugation at 7649 x g for 20 min at 8 °C. Supernatant was collected and aliquoted for storage at −70 °C until use.

### Collection of buffy coat cells

Approximately 6 mL of whole blood was collected in EDTA tubes from a captive Southern African Warthog (*Phacochoerus africanus sundevallii*) by the Potawatomi Zoo of South Bend Indiana and shipped overnight with ice packs to Plum Island Animal Disease Center in Greenport, New York. Samples were evaluated upon arrival and appeared slightly hemolyzed. Buffy coat cell extraction was performed as previously described [21] and approximately 1.2 × 10^7^ cells were obtained. Cells were resuspended in cryopreservation media (90% FBS and 10% DMSO) at a concentration of roughly 1.0 × 10^6^ cells/mL and stored at −150 °C until use in assays. Warthog red blood cells (RBCs) were diluted to 25% in DPBS then stored at 4 °C for usage in hemadsorption assays. For control purposes, buffy coat cells from domestic swine blood were obtained and cryopreserved as previously described [21].

### Collection of swine bone marrow

Swine bone marrow cells were collected by harvesting the femur of an 8-week-old swine that was humanely euthanized. For collection, the femur was bisected and a cell canula was used to disrupt bone marrow. Twenty-five mL of cell harvest media (RPMI-1640 with HEPES/L-glutamine and 1% antibiotic/antimycotic) was pipetted into the femur to resuspend the cells and subsequently collected in 50 mL conical tubes. Resuspended cells were centrifuged at 1700 x g for 10 min at 4 °C to pellet the cells and discard the supernatant. Cells were resuspended in 3 mL of ammonium chloride solution (STEMCELL™, 07850), briefly vortex-mixed, and incubated on ice for 5 min with gentle swirling to lyse carryover RBCs. After incubation, 10 mL of wash media (1x DMEM, 1% antibiotic/antimycotic) was added to stop lysis. Resuspended cells were then strained through a 100 μm cell strainer. Flow through was subjected to centrifugation at 1700 x g for 10 min at 4 °C to re-pellet cells. Collected cells were subjected to repeated ammonium chloride solution incubation and washing until free of RBC contamination. Resultant bone marrow cells were resuspended in cryopreservation media at a cell concentration of 1 × 10^7^ cells/mL, aliquoted, and stored at −150 °C until use.

### ASFV permissiveness testing

For ASFV permissiveness testing, cryopreserved warthog buffy coat cells were thawed and resuspended in M∅ base media (49% RPMI, 30% L929 conditioned media, 20% gamma-irradiated FBS, 1% antibiotic/antimycotic, and 0.1% gentamicin) and seeded in 48-well cell culture plates (Cyto-One^®^) at a seeding density of 2.5 × 10^5^ cells/well. Cells were incubated in 200 µL of M∅ base media per well at 37 °C with 5% CO_2_ for 24 h. After incubation, unattached cells were gently removed by rinsing with incubation media and fresh M∅ base media applied to each well prior to infection with ASFV.

Permissiveness of cells to three different ASFV isolates, Georgia ‘07, Tengani ’62 and Lisbon ’60, and one attenuated vaccine candidate, ASFV-G-ΔMGF [17], were evaluated. In addition to cell culture derived virus, Georgia’07 spleen homogenate from infected domestic swine was also tested. All viral stocks were titrated on swine PBMCs in 96-well Corning^®^ Primaria™ cell culture plates at a seeding density of 1 × 10^6^ cells/well to obtain the 50% hemadsorbing dose (HAD_50_/mL) values prior to use in experiments. Prior to application of diluted virus stocks, seeded PBMCs were allowed to differentiate in M∅ base media overnight. Following overnight incubation of PBMCs, virus stocks were diluted from − 1- to 12-fold in M∅ base media. Diluted RBCs were added to each viral dilution for a final ratio of 1:50 RBCs to diluted virus. A volume of 100 µL of the final diluted virus stocks with RBCs was applied to each well within the 96-well PBMC plates for a final volume of 200 µL per well. At 7- days post-infection (dpi) wells were scored for the presence of hemadsorption to determine titers.

Both swine and warthog derived buffy coat cells were infected with up to five different MOIs: 0.05, 0.10, 1.0, 2.0, and 5.0. Cells were evaluated for the formation of rosettes at 2-, 3-, and 8- days post infection utilizing a 1:10 dilution of both swine and warthog red blood cells (RBC). Supernatant was harvested at 7 dpi for determination of viral titer utilizing the HAD_50_ assay, as described above, and calculated using the Reed-Muench method [23]. Due to limitations on availability of warthog buffy coat cells, infection with Georgia ’07 virus and Georgia ’07 spleen homogenate was not performed at an MOI of 5 and limited infections were done at an MOI of 2.0. Results from limited 2.0 MOI infections are available in the Supplemental Dataset but not included in figures or analysis due to lack of statistical replicates.

## Results and discussion

### Confirmation of hemadsorption with heterologous sourced cells

**Fig. 1:**
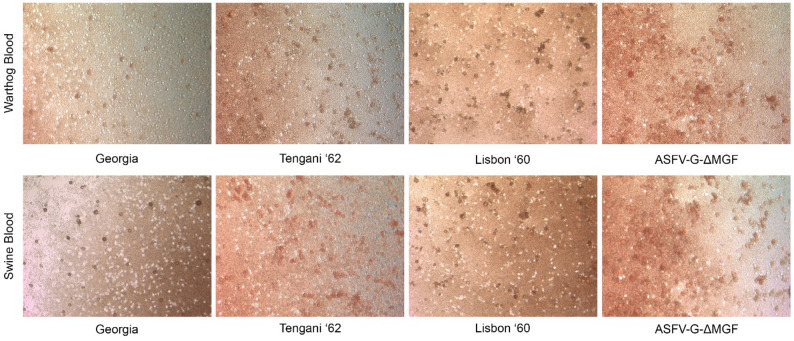
Swine PBMCs infected with three different ASFV isolates and one attenuated virus vaccine candidate. At 2 days post-infection cells had either African Warthog or Domestic Swine sourced RBCs applied and demonstrated hemadsorption

Using swine RBCs to test for hemadsorption following ASFV infection is long established in the field and blood from wild Suidae species has also been found to be useful for hemadsorption on homologous sourced cells [8]. Due to limited availability of RBCs from wildlife, we evaluated the use of RBCs in a heterologous fashion for hemadsorption following ASFV infection. Swine PBMCs infected with multiple isolates of ASFV were found to demonstrate hemadsorption following application of RBCs, regardless of swine or warthog origin, Fig. 1, validating that RBCs of either species produces hemadsorption in infected cells.

### Usage of warthog buffy coat cells for ASFV replication

**Fig. 2:**
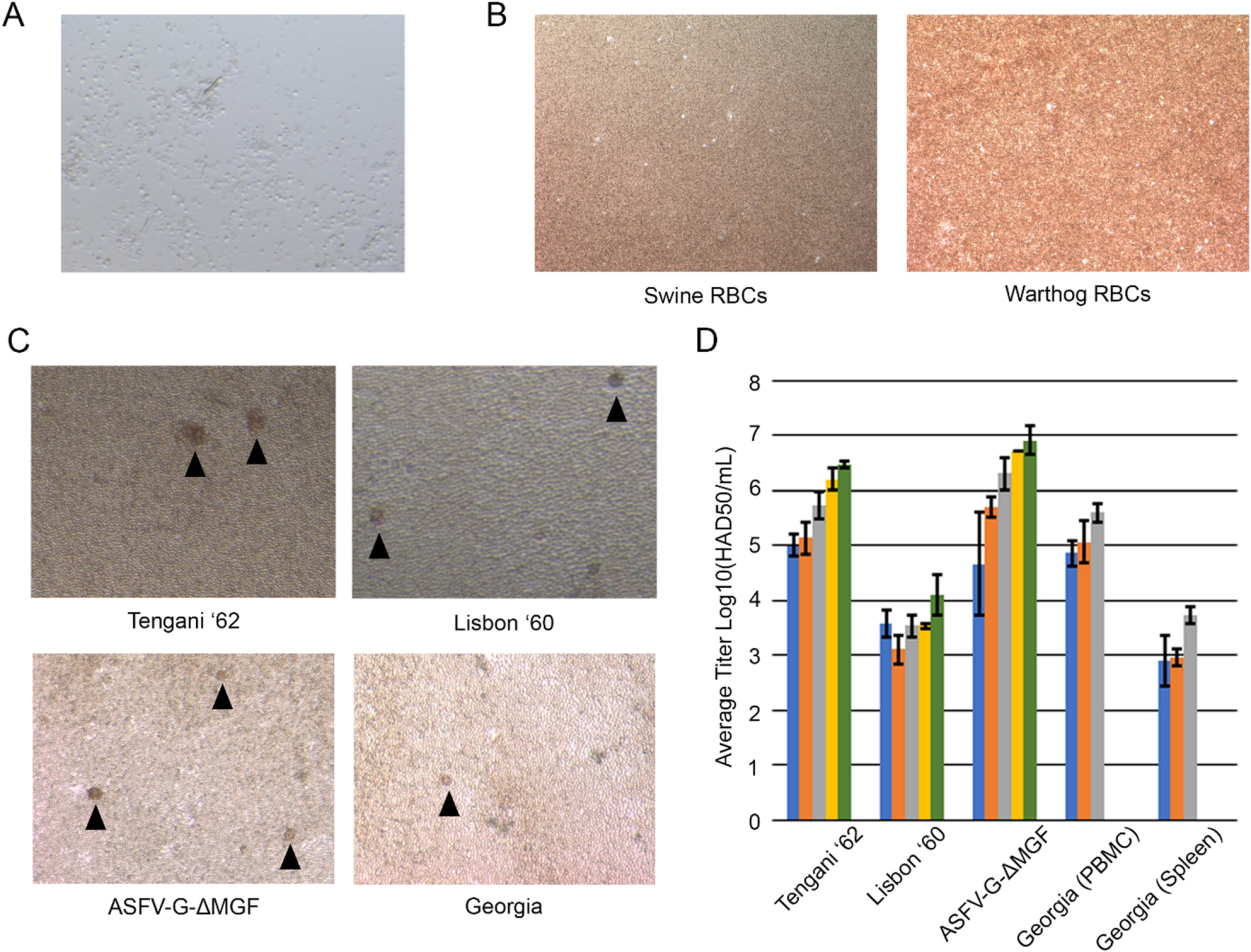
(**A**) Cryopreserved WBC cells retained viability and adhered to cell culture plates. (**B**) In the absence of virus, no hemadsorption is present with WBC cells regardless of RBCs utilized. (**C**) When WBC cells are infected with different isolates of ASFV hemadsorption is detectable in all isolates confirming ASFV infection; black triangles mark incidents of hemadsorption. (**D**) Final titer obtained at 7 dpi demonstrates diversity amongst different isolates and MOIs, 0.05 (blue), 0.01 (orange), 1.0 (gray), 2.0 (yellow), and 5.0 (green)

Cell seeding densities previously utilized with swine buffy coat cells [21] were used to validate cryopreserved warthog-sourced buffy coat cells susceptibility to ASFV. Viability and attachment of warthog buffy coat (WBC) cells was confirmed, Fig. 2A. To ensure that any observed hemadsorption was not an artifact of utilizing WBC cells, RBCs from both domestic swine and warthog were applied with no spontaneous hemadsorption observed in the absence of ASFV infection, Fig. 2B.

To test the ability of ASFV to replicate in warthog sourced buffy coat cells, up to five different MOIs: 0.005, 0.01, 1.0, 2.0, and 5.0, from five different sourced inoculums were applied to cells. Three inoculums were sourced from virus isolates passaged on swine PBMCs: Georgia, Tengani ’62, and Lisbon ’60. These viruses represent genotypes II, V, and I, respectively [2, 24], although revaluations of viral genomic diversity have recently proposed grouping Georgia and Tengani ’62 both into genotype II [25]. One inoculum originated from spleen homogenate of Georgia ‘07 experimentally infected swine. The final inoculum was an attenuated vaccine candidate, ASFV-G-MGF [17], passaged on swine PBMCs. These inoculums were chosen as surrogates for potential usages for WBC cells including evaluating passaged virus isolates, harvest of virus from infected tissues, and culturing of live attenuated vaccine candidates.

All tested viruses produced hemadsorption on WBC cells, Fig. 2C. WBC cells infected with MOIs of 0.05 and 0.1 predominantly had hemadsorption observed at 3 dpi whereas higher MOIs typically presented at 2 dpi. While hemadsorption was observed regardless of RBCs origin, swine RBCs provided a more definitive observation, likely related to the hemolysis of RBCs observed upon arrival. Final titrations of virus for infected cells at 7 dpi across multiple MOIs demonstrated a diversity among different isolates. Differences among viral titers from the lowest, 0.05 and 0.1, to highest, 2.0 and 5.0, MOIs are observed for Tengani ’62 and ASFV-G-MGF but are less pronounced for Lisbon ’60 and Georgia ‘07 inoculums, Fig. 2D.

### Evaluation of cryopreserved cells as an alternative to whole blood transport

While shipment of whole blood for buffy coat extraction was successful, Fig. 2, it risked sample hemolysis and subsequent detriment to cell quality relative to samples not subjected to overnight storage and transportation [21]. An alternative to shipping whole blood is transportation of cryopreserved cells processed from whole blood at the collection location. To confirm that cryopreserved cells could be utilized in this manner we harvested both swine buffy coat and bone marrow cells then transported them on dry ice overnight for ASFV susceptibility testing.

**Fig. 3:**
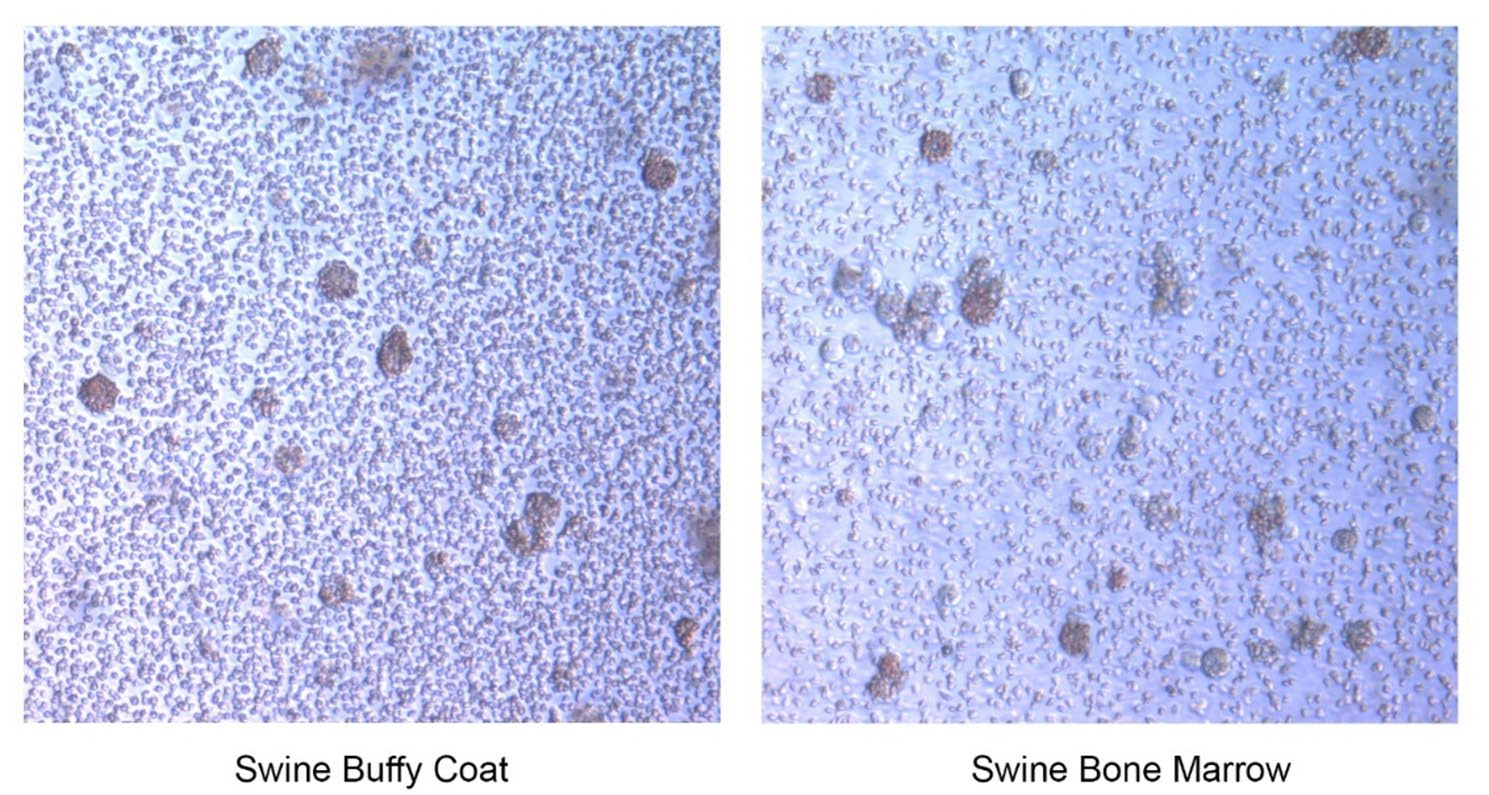
Swine buffy coat and bone marrow cells retain ability to support ASFV growth after being shipped in a cryo-preserved state

Both cell types recovered from cryopreservation and upon application of ASFV, demonstrated hemadsorption, Fig. 3. The usage of transported cryopreserved cells avoids the risk of hemolysis found with transported whole blood, providing benefits over the transport of whole blood.

## Conclusion

Understanding the dynamics of ASFV, and attenuated vaccine strains, in primary cells of different species can provide insight into broader species susceptibility and viral genomic stability. This is particularly relevant for critically endangered species, such as the Visayan warty pig, with documented susceptibility to African Swine Fever [8]. The work presented utilized a partner organization with access to African Warthogs, known to be ASF susceptible, to provide whole blood for extraction of buffy coat cells to evaluate ASFV in wildlife sourced cells. This methodology maximizes utilization of existing wildlife resources, such as zoological parks, with high containment laboratories working on animal and One-Health initiatives. Through usage of transported whole blood or cryopreserved primary cells, future research can evaluate ASFV in wildlife species in vitro, such as performing viral genomic stability studies, both with outbreak strains and attenuated vaccine strains. These results would provide critical data for evaluation of the risk of vaccination in case of an ASF outbreak.

## Supplementary Information

Supplementary Material 1.

## Data Availability

Data generated during this study is available in supplementary material.
